# Apoptosis Detection in Retinal Ganglion Cells Using Quantitative Changes in Multichannel Fluorescence Colocalization

**DOI:** 10.3390/bios12090693

**Published:** 2022-08-28

**Authors:** Xudong Qiu, Seth T. Gammon, James R. Johnson, Federica Pisaneschi, Steven W. Millward, Edward M. Barnett, David Piwnica-Worms

**Affiliations:** 1 Department of Cancer Systems Imaging, University of Texas MD Anderson Cancer Center, Houston, TX 77030, USA; 2 Mallinckrodt Institute of Radiology, Washington University in St. Louis, St. Louis, MO 63130, USA; 3 Department of Ophthalmology & Visual Sciences, Medical College of Wisconsin, Milwaukee, WI 53226, USA

**Keywords:** apoptosis, retinal ganglion cell, dual-fluorophore peptide sensor, fluorescence resonance energy transfer, NMDA, anterograde axonal transport, glaucoma, multispectral detection, fluorescence colocalization

## Abstract

KcapTR488 is a dual-fluorophore peptide sensor for the real-time reporting of programmed cell death by fluorescence imaging. KcapTR488 contains a nuclear localization sequence (NLS) conjugated with Texas Red, a caspase-cleavable sequence (DEVD), and a C-terminus conjugated to Alexa Fluor 488 (AF488). The synthesis and preliminary evaluation in cellulo of KcapTR488 for monitoring cell death by fluorescence imaging has been previously reported, but its utility in vivo has yet to be tested or validated. Herein, in vitro solution experiments verified the intramolecular fluorescence resonance energy transfer (FRET) between the two fluorophores and enabled a quantitative analysis of enzyme rates and selectivity. The sensor delivery kinetics in live rat models were quantified by ex vivo fluorescence microscopy. Studies in healthy control retinas demonstrated that KcapTR488 concentrated in the nucleus of retinal ganglion cells (RGC), with a strong colocalization of red and green fluorescence signals producing robust FRET signals, indicating an intact reporter. By contrast, using an acute but mild NMDA-induced retinal injury model, dual-color confocal ex vivo microscopy of cleaved KcapTR488 identified sensor activation as early as 2 h after injection. Quantitative changes in fluorescence colocalization were superior to changes in FRET for monitoring injury progression. Longitudinal monitoring revealed that the NLS-Texas Red fragment of the cleaved sensor moved out of the cell body, down the axon, and exited the retina, consistent with anterograde axonal transport. Thus, KcapTR488 may be a powerful tool to study RGC death pathways in live preclinical models of glaucoma.

## 1. Introduction

The initiation of apoptosis leads to the activation of initiator caspases (cysteine-dependent aspartate-specific proteases) 2, 8, 9, or 10, followed by the activation of effector caspases 3, 6, or 7, which commit cells to programed cell death [1,2,3]. Recently, KcapTR488, a dual-fluorophore peptide sensor, was developed to enable the multispectral detection of caspase activation through changes in fluorescence colocalization [4,5]. This reporter comprises three motifs: a D-amino acid permeation motif/nuclear localization sequence (NLS) coupled to the Texas Red fluorophore, followed by an L-amino acid caspase-cleavable sequence (DEVD), and finally, a C-terminal L-cysteine amino acid coupled to the AF488 fluorophore [5,6]. An intact (inactivated) reporter qualitatively appears yellow and quantitatively demonstrates a strong correlation between the red and green fluorescence emission channels. When effector caspases are activated, KcapTR488 can then be cleaved by caspases 3/7 at the DEVD sequence. In neuronal cells in culture, the cleaved NLS-Texas Red fragment is enriched in the nucleus, while the AF488-lysine conjugate remains primarily cytosolic [5]. Similar reporters utilize changes in subcellular distribution from the cytoplasm to the nucleus but require morphologic quantification and are difficult to automate [7]. By using KcapTR488, the change in subcellular color localization may enable the rapid and precise measurement of cell death on diverse instrumentation platforms using standard multichannel microscopes.

Retinal ganglion cells (RGCs) are retinal neurons with cell bodies and axons located in the inner retina, adjacent to the vitreous humor, whose axons comprise the optic nerve in vivo and extend into the brain [8]. The final fate of the KcapTR488 cleavage fragments in vivo remains to be resolved and may be fundamentally different compared to that observed in vitro or in cellulo. In one model, KcapTR488 is transported into RGCs by non-receptor-mediated endocytosis [5], and in contrast to neuronal cells in culture, once within the cytoplasm of the RGC cell bodies, caspase-mediated cleavage of the sensor enables the active transport of the permeation motif both into the nucleus and anterograde down the axon, leading to the loss of colocalization between Texas Red and Alexa 488.

While the KcapTR488 sensor has been tested in cultured cells, detailed spectral analysis, caspase selectivity, and behavior in vivo have yet to be validated. Herein, the potential FRET properties of the reporter were explored in vitro and leveraged to characterize the caspase selectivity of the reporter with purified caspases. The NMDA model of caspase induction was utilized to test KcapTR488 in vivo using fluorescence imaging analysis **ex vivo**. This NMDA model of RGC death has been utilized extensively to study earlier molecular beacons for the detection of RGC death in vivo and demonstrated the potential for the molecular imaging of RGC apoptosis in glaucoma [9,10,11,12,13]. Herein, we further demonstrated that KcapTR488 detected effector caspase activity in RGC apoptosis using both multispectral analysis and FRET ratio monitoring. KcapTR488 is selective for executioner vs. initiator caspases and inflammatory caspases in vitro, enabling the monitoring of RGC death in vivo using ex vivo multichannel confocal fluorescence microscopy at key time points in the process.

## 2. Materials and Methods

### 2.1. Animals

The Institutional Animal Care and Use Committee at the University of Texas MD Anderson Cancer Center approved all animal experiments, which adhere to the Association for Research in Vision and Ophthalmology (ARVO) Statement for the use of animals in ophthalmic and vision research. Male Brown Norway rats weighing approximately 200 g each were purchased from Envigo.

### 2.2. Synthesis of Cell-Penetrating Peptide Probe KcapTR488

KcapTR488 was synthesized as previously reported [5,10,12]. Solid-phase N-α-Fmoc chemistry was applied to synthesize the core peptide (Tufts University Peptide Synthesis Core, Boston, MA). The core peptide (Kcap) was an all-D peptide containing the SV40 TAg NLS (KKKRKV). The NLS was linked to a caspase cleavage sequence consisting of L-amino acids (KDEVD) and a C-terminus cap (APC). Texas Red succinimidyl ester (Thermo Fisher Scientific, Waltham, MA, Cat#T20175) was conjugated via an NHS ester reaction to the lysine N-terminus of the DEVD sequence that was deprotected from the Dde group. Alexa Fluor 488 C5 maleimide (Thermo Fisher Scientific, Waltham, MA, Cat#A10254) was conjugated to the C-terminus cysteine residue via a maleimide reaction. Purification was performed via preparative HPLC using a Luna 5 μm C18(2) LC Column (150 × 21.2 mm; Phenomenex, Torrance, CA) and the following gradient: A—water (0.1% TFA), B—acetonitrile (0.1% TFA); B%: 0→42 in 10 min, 42 for 10 min, 42→90 at a wavelength of 460 nm (rt = 13.7 min). After lyophilization, KcapTR488 was obtained as a blue solid. The purity was assessed by analytical HPLC (column: Alltech Adsorbosphere C18 5 μm, 250 mm. method: A—water (0.1% TFA); B—acetonitrile (0.1% TFA); B% 5→40 in 15 min, 40 for 15 min, 40→95 in 3 min, 95→5 in 1 min) at 600 nm, and identity was confirmed by MALDI mass spectrometry (m/z 3141.3 [MH+]; calc 3140.3).

### 2.3. In Vitro Caspase Activity Assay

Prior to the biochemical analysis, the linearity and reproducibility of the in vitro assays were determined (Figure S2). Each concentration was tested in triplicate, and the test–retest agreement was verified with the indicated concentration of peptide.

The highly reproducible and linear response of the assay enabled the use of an overdetermined spectral unmixing algorithm to quantify the amount of product produced in vitro (Figure S2).

To optically characterize the Texas Red and Alexa Fluor 488 intramolecular interactions in KcapTR488, 5 µM of KcapTR488 and Alexa Fluor 488 were dissolved in 1 X caspase3 assay buffer (Cat#A0219, Sigma-Aldrich, St. Louis, MO, diluted in MilliQ water from 10 X assay buffer). Texas Red was initially dissolved in DMSO and then diluted to 5 µM in 1 X caspase 3 assay buffer. Caspase 3 (Cat#CASP3F, Sigma-Aldrich, St. Louis, MO) was added to one set of KcapTR488 solution at a final concentration of 15 nM. Assays were conducted in triplicate in 96-well, clear-bottomed, clear-walled plates at a final volume of 100 µL. All samples were arranged in a pattern, leaving at least one empty well between adjacent samples. Assays were performed in a Synergy H4 microplate reader (BioTek; excitation 480 nm ± 4.5 nm, emission scans from 505 nm to 700 nm; excitation 540 nm ± 4.5 nm, emission scans from 565 nm to 700 nm; emission scans were stepped at 3 nm intervals, gain 80, read height 8 mm; temperature 37 °C).

For the Km and Kcat studies, caspase assays were performed at KcapTR488 concentrations ranging from 0.06 µM to 8 µM for caspase 1 (25 nM), 3 (0.14 nM), 7 (5.4 nM), and 8 (15 nM). Assays were performed at 37 °C. Blanks and zero-added enzyme controls were included for each experiment. The time series data were converted into the concentration of product generated per unit time via Figure S2 (Mathematica) on a per-well basis. The initial rates were calculated on a per-well basis, defined as [product] ≤ 10% of initial concentration of starting material up to 1.5 h maximum. The initial rates were then normalized to the sum of saturable enzyme activity + nonspecific hydrolysis (GraphPad Prism) for each enzyme. The concentration dependence for each enzyme was fit three independent times.

### 2.4. Intravitreal Injections

Intravitreal injections were performed as previously described [12,13]. Briefly, rats were anesthetized by isoflurane inhalation. NMDA and/or KcapTR488 were injected into the vitreous under stereomicroscope guidance. A U-100 29-gauge 1/2-inch insulin needle was used to create a small sclerotomy site at approximately 1 mm posterior to the limbus in the nasal quadrant. A 33-gauge 1-inch blunt metal needle on a 2.5 µL Hamilton syringe (Hamilton Company, Reno, NV) was inserted into the posterior chamber through the sclerotomy to deliver injectate (either 2 µL of 25 mM NMDA or 2.5 µL of 100 µM KcapTR488) into the vitreous. Injections were performed slowly over 3 min. Neomycin/Polymyxin B/Bacitracin Ophthalmic Ointment was applied topically to the injection site after each intravitreal injection.

### 2.5. Whole-Mount Retinal Tissue and Retina Cross Section Preparation

Rats were sacrificed in a CO_2_ chamber. A transcardiac perfusion of 1000 units of heparin in 10 mL of 0.01 M PBS was followed by 4% paraformaldehyde in 10 mL of 0.01 M PBS. Whole eyes were harvested and fixed in 4% paraformaldehyde overnight at 4 °C. Following three 15 min washes in 0.01 M PBS, the retinas from the harvested eyes were dissected under a microscope. Whole-mount retinal tissue was prepared on slide glasses. For the preparation of retina cross sections, dissected retinas were further processed for cryoprotection. Retinas were washed in order with 5%, 10%, and 15% sucrose/0.1 M PBS solutions for 30 min and a 20% sucrose/0.1 M PBS solution overnight at room temperature. Retinas were placed into a 2:1 volume mix of 20% sucrose/OCT, respectively, for an hour and pure OCT for another 30 min. Retinas were then embedded in a cryomold with fresh OCT and slowly frozen on dry ice. Retinas were stored in a −80 °C deep freezer in zip lock bags until they were sectioned into 12 µm vertical cross sections.

### 2.6. Antibody Staining and TUNEL Labeling

For immunohistology staining, whole-mount retinas or retinal cross sections were permeabilized in 0.5% Triton X in 0.01 M PBS for 15 min at room temperature, then switched to a blocking solution of 0.1% Triton X and 1% BSA in 0.01 M PBS for 2 h at room temperature. For RGC marker staining, a primary anti-RNA-binding protein with a multiple splicing (anti-RBPMS) antibody (Millipore Sigma, Burlington, MA, USA), Cat#ABN1362) was diluted to 1:200 in blocking solution and added to the tissue for incubation at 4 °C for 4 days. After primary antibody incubation, tissues were washed three times for 15 min each in the blocking solution. The secondary antibody Alexa Fluor 647 (Thermo Fisher Scientific, Waltham, MA, USA), Cat#A-21245) at a 1:400 dilution in 0.01 M PBS was then added to the tissue and incubated at 4 °C overnight. RBPMS was found to be exclusively expressed in RGCs and displaced RGCs (dRGCs) and was therefore utilized as an RGC marker [14]. For amacrine cell marker staining [15,16], a primary anti-choline acetyltransferase antibody (anti-ChAT, Millipore Sigma, Burlington, MA, USA, Cat# AB144P) at a 1:200 dilution was prepared and added to the tissue for incubation for 4 days at 4 °C. After three 15 min washes in blocking solution, the secondary antibody Alexa Fluor 647 (Thermo Fisher Scientific, Waltham, MA, Cat#A-21447) at 1:400 was added to the tissue for overnight incubation at 4 °C. After secondary antibody incubation, the tissue was washed in 0.1% Tween 20 in 0.01 M PBS three times for 15 min each. Retinal tissue was mounted on slide glass with prolong Gold antifade mountant (Thermo Fisher Scientific, Waltham, MA, USA, Cat# P36935) with or without DAPI for confocal microscopic imaging. TUNEL staining was performed on 12 µm retinal cross sections using the manufacturer’s protocol (Click-iT Plus TUNEL Assay, Thermo Fisher Scientific, Waltham, MA, USA, Cat#C10619).

### 2.7. Confocal Microscope Imaging

Confocal microscopic images were acquired under an Olympus FLUOVIEW FV1000 confocal laser scanning microscope using a PLAPO 60 X oil objective lens at a 12 µs/pixel sampling speed. For the detection of the sensor KcapTR488 signal, laser intensities were adjusted accordingly to minimize saturation and maximize positive signal pixels in both the FITC and Texas Red channels.

### 2.8. Confocal Image Analysis

The colocalization of fluorophores AF488 and Texas Red was accessed by ImageJ plugin JACoP (Just Another Colocalization Plugin). JACoP is a set of colocalization-calculating tools and includes Manders’s overlap coefficient, which we used to calculate the AF488 overlap with Texas Red under different conditions. The coefficient varies from 0 to 1, with 0 indicating nonoverlapping images and 1 reflecting 100% colocalization between images. When importing paired AF488 and Texas Red images to the plugin, thresholds of both images were calculated by adding three times the StdDev to the mean intensity of the selected background area. When selecting the background area, image edges were avoided. M1 and M2 values served as indicators of the colocalization of AF488 and Texas Red signals. M1 is defined as the ratio of the ‘summed intensities of pixels from the green image for which the intensity in the red channel is above zero’ to the ‘total intensity in the green channel’, and M2 is defined conversely for red. Therefore, M1 (or M2) is an indicator of the proportion of the green signal coincident with a signal in the red channel divided by its total intensity [17].

In addition, we also calculated the fluorescence resonance energy transfer (FRET) ratio in the cell body to assess the proximity interactions between fluorophores AF488 and Texas Red within KcapTR488. For confocal microscopic scanning, excitation was set at 488 nm and emission was set at either 500–550 nm (FITC signal) or 595–645 nm (FRET signal). The FRET ratio equals the FRET signal divided by the FITC signal on a per-pixel basis. Cell body ROIs were then selected in ImageJ by creating a mask based on size and circularity. The average FRET ratio for each cell body, as defined in the mask, was quantified in the FRET image. The same process and values were utilized for all images.

## 3. Results

### 3.1. In Vitro Characterization of KcapTR488

**Figure 1:**
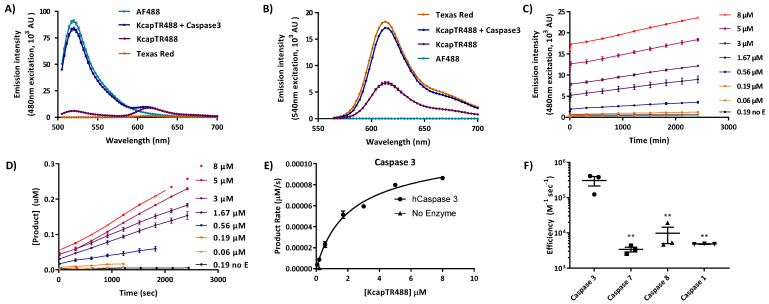
Changes in FRET between fluorophores in KcapTR488 reporter detected caspase activity in vitro. Emission scan for AF488, KcapTR488 with Caspase 3, KcapTR488, and Texas Red at excitation wavelengths of 480 nm (**A**) and 540 nm (**B**) revealed two-way FRET between AF488 and Texas Red. The emission peak of KcapTR488 at 520 nm increased 13.92 ± 0.04 (SD) fold when caspase 3 was added (**A**). The addition of caspase 3 also increased KcapTR488 emission at 613 nm by 2.54 ± 0.13 (SD) fold at the excitation wavelength of 540 nm (**B**). Caspase 3 (0.14 nM) was incubated with various concentrations of substrate KcapTR488, and the emission at 520 nm (excitation at 480 nm) was recorded over time (**C**). The products (**D**) and initial rate (**E**) of enzymatic caspase 3 cleavage were calculated based on the nonlinear fitting of a concentration of cleaved product to the observed emission ratio at each time point. Caspases 7, 8, and 1 were also tested using the same experimental design. The catalytic efficiency, Kcat/Km, was calculated for all four caspase enzymes (**F**), which indicated that caspase 3 was the preferred enzyme for KcapTR488. ** represents *p* < 0.01.

To characterize the potential optical interaction between the two fluorophores on KcapTR488, we investigated the florescent emission spectra of solutions of AF488 or Texas Red alone and of the dual-labeled KcapTR488 sensor at identical concentrations, with and without caspase 3 enzyme in the assay buffer, at excitation wavelengths of either 480 nm (Figure 1A) or 540 nm (Figure 1B). Figure 1A shows the results of an emission scan at excitation wavelength of 480 nm for all four samples. Intact KcapTR488 had emission peaks at both 520 nm and 613 nm, where the ratio between the two emissions (520 nm/613 nm) was 0.69 ± 0.003 (SD). Texas Red alone had a detectable emission peak at 613 nm that was only 12% ± 0.08% (SD) of the KcapTR488 emission intensity at 613 nm, while AF488 alone had an emission peak that was 15-fold higher than KcapTR488. The stronger emission intensity of intact KcapTR488 at 613 nm and lower emission intensity at 520 nm were consistent with fluorescence resonance energy transfer (FRET), occurring from AF488 to Texas Red upon excitation at 480 nm. If FRET was responsible for the increased emission at 613 nm, caspase-mediated cleavage of the DEVD sequence between the fluorophore pair should result in a greater intermolecular distance between them, eliminating FRET and restoring the emission intensities at 520 nm and 613 nm. Consistent with this, the addition of caspase 3 to KcapTR488 resulted in an emission at 520 nm that was comparable to AF488 alone with a 13.92 ± 0.04 (SD) fold increase compared to KcapTR488 alone. Indeed, caspase cleavage was independently validated by HPLC analysis and, as expected, yielded a positive nonlinear correlation with the FRET signal on a per-sample basis (r = 1; *p* = 0.0028, two-tailed Spearman test) (Figure S3).

Figure 1B shows results of the emission scan at 540 nm excitation for all four samples. For KcapTR488 plus caspase 3, the peak emission intensity at 613 nm was comparable to Texas Red alone. A 2.54 ± 0.13 (SD) fold increase was noted at 613 nm compared to KcapTR488 alone, suggesting that in an intact KcapTR488, AF488 also had a quenching effect on Texas Red. As expected, AF488 alone did not show detectable emissions at 540 nm excitation. This indicated that cross talk between pairs of excitation and emission filters should be negligible when using a high-quality multichannel fluorescence microscopy instrument with dual-excitation and emission pairing.

Therefore, we confirmed that the spectral response at key excitation and emission pairs could be used as an indicator of KcapTR488 cleavage by recombinant caspases. Figure 1C shows representative results of caspase 3 cleavage of the KcapTR488 sensor at different concentrations over time, and the emission intensity was converted to product formation, as shown in Figure 1D. Next, the rate of caspase-3-mediated product generation was calculated as a function of sensor concentration (Figure 1E). The calculated catalytic efficiency of caspases 1, 3, 7, and 8 are shown in Figure 1F (note log scale). Table 1 summarizes the Km, Kcat, and efficiency values for all four tested caspase enzymes for the KcapTR488 sensor. Indeed, the Km for caspase 3 was within 2 μM of the Km deduced for previous permeation-peptide-coupled caspase-activated molecular beacons [11,18].

### 3.2. Immunohistochemistry Staining Confirmed Uptake of Intravitreally Injected KcapTR488 in Rat RGCs and Displaced Amacrine Cells

**Figure 2:**
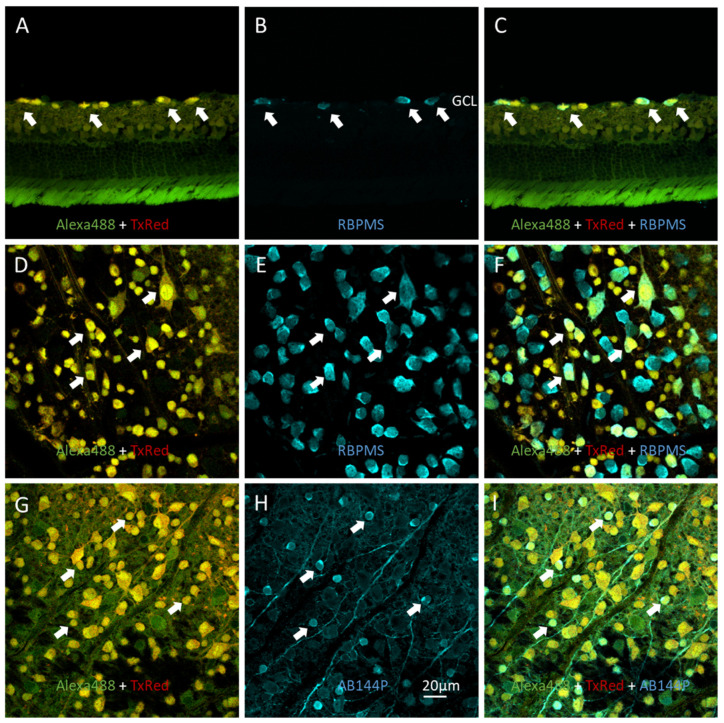
Immunohistochemistry staining on control healthy rat retinas confirmed uptake of intravitreal injected KcapTR488 in RGCs and displaced amacrine cells. Immunofluorescence staining in retina cross sections (**A**–**C**) and flat mount (**D**–**F**) retinas for RGC marker RBPMS (**B**,**C**,**E**,**F**) showed uptake of reporter KcapTR488 by RGCs. Immunohistochemistry staining of amacrine cell marker AB144P in flat mount retina (**G**–**I**) showed uptake of reporter KcapTR488 by displaced amacrine cells. Arrows indicate sets of RBPMS or AB144P-positive cells that had KcapTR488 uptake labeled as yellow in control retinas. Scale bar (20 µm) in (**H**) applies to (**A**–**I**). Ganglion cell layer (GCL).

RGCs, which are in close proximity to the vitreous in the ganglion cell layer (GCL), preferentially accumulate intravitreally delivered probes [5,10,11,12]. In the rodent GCL, it has been estimated that 50% of the cell bodies are displaced amacrine cells, 40% are RGCs, and astrocytes comprise the remainder [19,20]. To determine whether probe uptake in the GCL was specific to RGCs, we performed neuronal marker immunostaining on retinas (Figure 2) to confirm the identities of cells displaying sensor uptake.

Figure 2A–F show the results of antibody staining against RNA-binding protein with multiple splicing (RBPMS) in retinal cross sections (Figure 2A–C) and flat mount retinas (Figure 2D–F). RBPMS is a unique RGC marker [14,15]. Figure 2A,D are superimposed images of FITC and Texas Red spectral windows focused on the GCL from healthy retinas, demonstrating the distribution of KcapTR488. Figure 2B,E demonstrate RBPMS antibody staining. RBPMS-positive cells were noted in the GCL, as expected. An overlay of the sensor labeling image onto the RBPMS staining image confirmed that RGCs accumulated the intravitreally delivered probe (Figure 2C,F). Furthermore, the results also suggested the presence of RBPMS-negative, probe-positive cells, likely representing displaced amacrine cells in the GCL.

Figure 2G–I show the results of anti-choline acetyltransferase (ChAT) antibody (AB144P) staining in flat mount retina. ChAT is a marker for a group of retinal amacrine neurons [15,16]. Figure 2G is a superimposed image of FITC and Texas Red spectral windows focused on the GCL in healthy retinas loaded with KcapTR488. AB144P staining is shown in Figure 2H, where AB144P/ChAT-positive cells in the GCL generally had small cell bodies with large nuclei. A three color overlay (Figure 2I) confirmed the colocalization of ChAT staining and KcapTR488 labeling, further indicating that displaced amacrine cells located in the GCL also took up KcapTR488 following the intravitreal injection.

### 3.3. In Vivo Characterization of KcapTR488 Using Ex Vivo Fluorescence Microscopy

**Figure 3:**
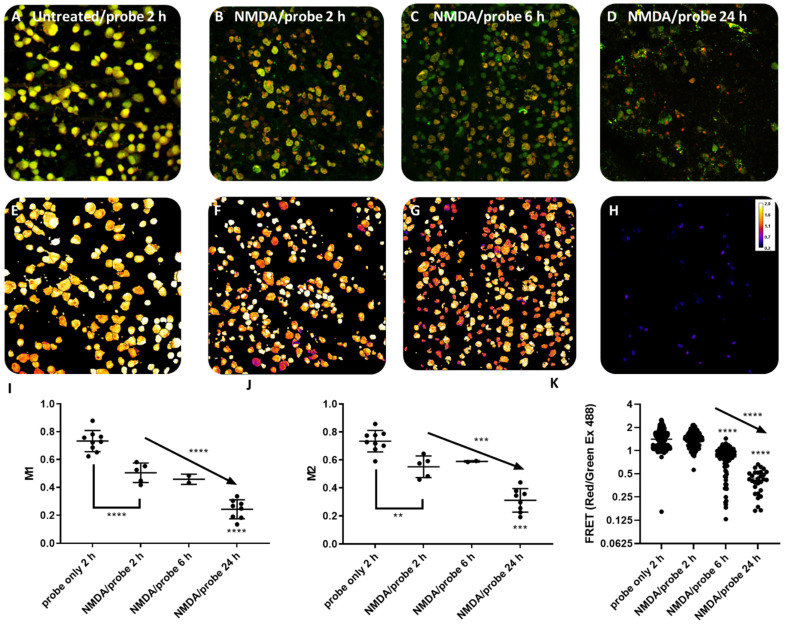
KcapTR488 detected apoptotic cell death in vivo quantitatively. Confocal images of KcapTR488 uptake in GCL cells from flat mount rat retinas showed a distinct labeling pattern under different treatment overlays of the AF488 signal (Green) and Texas Red (Red) (**A**–**D**) and distinct cell body FRET (**E**–**H**). Reporter-only control eyes were intravitreally injected with KcapTR488 for two hours (**A**,**E**). GCL cells (yellow) confirmed the reporter delivery and the colocalization of red and green in the healthy retina, and FRET/green ratios were high and comparable to the values found in vitro. Experimental eyes were treated with 50 nmol NMDA for 24 h and subsequently intravitreal injection of KcapTR488. Eyes were removed after 2 h (**B**,**F**), 6 h (**C**,**G**), and 24 h (**D**,**H**). The predominantly green cells in (**B**,**C**) indicated that caspase activation occurred in the cells with axonal transport and the removal of the cleaved NLS-Texas Red fragment out of the cell body, while AF488 stayed in the cell body. By 6 h, GCL cells had a more prominent red component in the cell body (**C**), which suggested a slowdown of NLS-Texas transport out of the cell body while retaining nuclear import. By 24 h (**D**), almost all cells had green cell bodies, and red-enriched nucleus labeling, indicating a near-complete loss of axonal transport while maintaining nuclear import. Data from whole fields were quantified for changes in two channel overlap with AF488 (green signal) and Texas Red (red signal) under ImageJ JACoP plugins (**I**,**J**). FRET was quantified on a per-cell-body basis; each datapoint represents an individual cell body (**K**). **** represents *p* < 0.0001, *** represents *p* = 0.001, ** represents *p* = 0.01. Arrows indicate the test for trend after the ANOVA analysis.

To test the hypothesis that KcapTR488 can detect apoptosis in vivo, KcapTR488 was delivered via intravitreal injection into rat eyes undergoing NMDA-induced RGC death. Figure 3A–D are representative confocal microscope images focused on the GCL from isolated flat mount retinas with or without NMDA pretreatment. The panels present superimposed images of FITC and Texas Red spectral windows to verify the colocalization of the fluorophores AF488 and Texas Red, respectively.

In a healthy retina (no NMDA) from an animal sacrificed 2 h after KcapTR488 sensor injection (Figure 3A), there was robust uptake in GCL cells. The reporter was concentrated in cell nuclei with evenly distributed yellow signal, indicating the colocalization of AF488 and Texas Red fluorophores, consistent with intact reporter. However, following 24 h of NMDA pretreatment, both yellow- and green-labeled cells in the GCL were noted within the same image 2 h after sensor injection (Figure 3B). The green-labeled cells in superimposed FITC and Texas Red spectral windows indicated fluorophore separation and delocalization, consistent with reporter cleavage by activated effector caspases. While not an intent of the study, no overt acute toxicity was observed, as expected from similar Kcap-based reporters [5].

In a retina pretreated with NMDA and the animal sacrificed 6 h after reporter injection, yellow- and green-labeled cells in the GCL were noted (Figure 3C). However, the qualitative distribution of the fluorophores appeared to be changing over time. The yellow- and green-labeled cells showed a more prominent red component compared to labeled cells from animals sacrificed 2 h after reporter injection (Figure 3B). Most yellow-labeled cells in Figure 3C demonstrated cytoskeletal fragmentation consistent with cellular degeneration.

Finally, Figure 3D shows a retina following 24 h of NMDA pretreatment with the animal sacrificed 24 h after reporter injection. GCL cells labeled dark red in nucleus-like structures and green in the cytosol were noted. This delocalization pattern of fluorophore AF488 and Texas Red in apoptotic cell bodies recapitulates the early results from in vitro KcapTR488 studies published from our lab [5], wherein cultured cells that had been preloaded with KcapTR488 were then treated with 10 µM ionomycin and showed AF488 in the cytosol but concentrated Texas Red in the nucleus.

The quantitative analyses of fluorophore colocalization and FRET under various conditions are summarized in Figure 3. Figure 3A–D were analyses based on Just Another Colocalization Plugin (JACoP) in ImageJ [17,21]. The colocalization indicators M1 in Figure 3I and M2 in Figure 3J indicated the colocalization of AF488 over Texas Red or conversely Texas Red over AF488, respectively. Both M1 and M2, calculated under various experimental conditions, indicated that NMDA treatment for longer times led to lower cellular colocalization of AF488 and Texas Red. Note that two-channel overlap experiments were not possible using an in vivo readout with the currently available instrumentation, but new multichannel and multiphoton visible ophthalmoscopes are currently under development [22].

In addition, FRET ratios were measured by the ratio of emission in the FITC spectral window over the Texas Red emission when excited by AF488 (Figure 3K). While the ratio displayed a large difference between the NMDA/24 h post reporter group and the other conditions, this ratio was not sufficiently sensitive to detect a loss of FRET at 2 h. The ratios achieved in vivo were consistent with those achieved in vitro.

Together, in healthy GCL neurons, these data demonstrated that the uptake of KcapTR488 was characterized by the nuclear localization of intact reporter. In the early stages of apoptosis, the cleaved NLS-Texas Red fragment subsequently exited the cell body in a subset of GCL neurons. In the late stages of apoptosis, the cleaved NLS-Texas Red fragment was not substantially transported down the axon and concentrated in the nucleus, while the AF488 fragment was evenly distributed in the cytosol. Therefore, KcapTR488 detected the progression of apoptotic cell death, as demonstrated by the changing subcellular distribution of fluorophores AF488 and Texas Red over time.

### 3.4. TUNEL Staining Corresponded to Apoptotic Cell Labeling by KcapTR488

**Figure 4:**
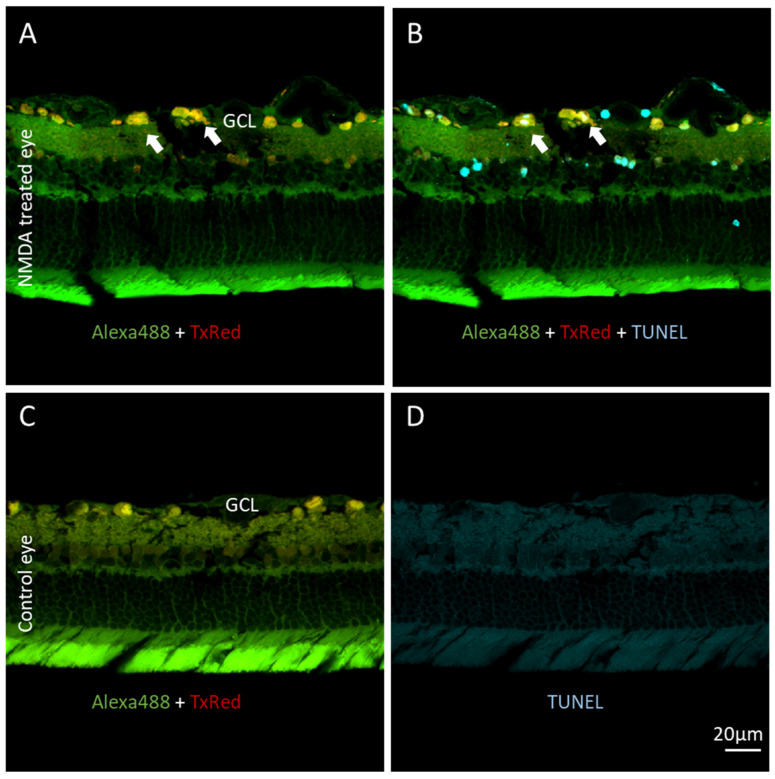
As previously reported, the mild NMDA model yields cell death in the GCL and KcapTR488 activation. GCL cells in vivo colocalize with TUNEL-positive labeling. Rat eyes were either treated with NMDA for 24 h (**A**,**B**) or untreated (**C**,**D**). Both eyes then had an intravitreal injection of KcapTR488 for 2–6 h and were excised. The arrows in images (**A**,**B**) point to the same set of representative cells that demonstrate the nuclear cytoplasmic separation of the red and green signal and positive TUNEL staining. In the untreated eye (**C**,**D**), GCL cells were evenly labeled in yellow and had no positive TUNEL labeling. Scale bar (20 µm) in (**D**) applies to (**A**–**D**). Ganglion cell layer (GCL).

Figure 4 shows the TUNEL staining results from an NMDA-pretreated retina loaded with KcapTR488 for 6 h (Figure 4A,B) and a control retina loaded with KcapTR488 for 2 h (Figure 4C,D). Figure 4A is a superimposed image of the FITC and Texas Red spectral windows. Arrows point to cells in the GCL with red-labeled nuclear-like structures surrounded by green-labeled cytosol. Again, the red-enriched nuclei indicated the separation of fluorophores AF488 and Texas Red due to activated effector caspases in the apoptotic cell body. As anticipated, the red-enriched nuclei colocalized with the TUNEL-positive signal, indicating that DNA fragmentation, an apoptotic hallmark downstream of effector caspase activation, had occurred in the apoptotic cells. Figure 4C is also a superimposed image of the FITC and Texas Red spectral windows from a control retina with KcapTR488 uptake only. TUNEL staining on the same retina did not result in a signal (Figure 4D), serving as a negative control. Together, the data shown in Figure 4 demonstrate that the model was responding to NMDA as expected and that KcapTR488 could detect apoptotic cell death in the retina in vivo by the use of a multispectral analysis of the subcellular localization of two fluorophores.

## 4. Discussion

Molecular beacons utilizing a fluorophore–quencher pair are optically silent in their intact state [21,23]. Such reporters do not provide information on cellular uptake without an orthogonal label and imaging modality. Therefore, a reporter that can confirm reporter delivery to the cell type of interest while also reporting on caspase activation, all with standard optical imaging channels, would be ideal. KcapTR488 was designed to leverage the multipurpose SV40 sequence KKKRKV that serves as a cell permeation motif, axonal transport signal, and nuclear localization sequence. The fluorophores flanking the DEVD sequence, Texas Red and AF488, were in close proximity and enabled multicolor readouts of caspase activity through changes in the subcellular localization of the fluorophores and also served to confirm reporter delivery to the target cell(s) of interest.

Unlike molecular beacons that rely on a single channel for characterizing enzyme efficiency, the FRET signal from KcapTR488 enabled the rapid evaluation of caspase specificity in vitro [5]. As expected, caspase 3 was the most efficient at cleaving the embedded DEVD sequence, while initiator caspases showed a minimal effect (Table 1). This effect was qualitatively and quantitatively similar to that observed with the previously characterized reporters TcapQ488 and TcapQ647 [18,24]. KcapTR488, in conjunction with two decades of experience with this class of peptides, confirmed that side-chain-linked fluorophore(s)/chromophore(s) coupled to the SV40 permeation motif provide a flexible platform that has been robust with a variety of fluorophore–quencher configurations. This platform is therefore likely to be amenable to continual advances in fluorophore–quencher pairs [25].

**Figure 5:**
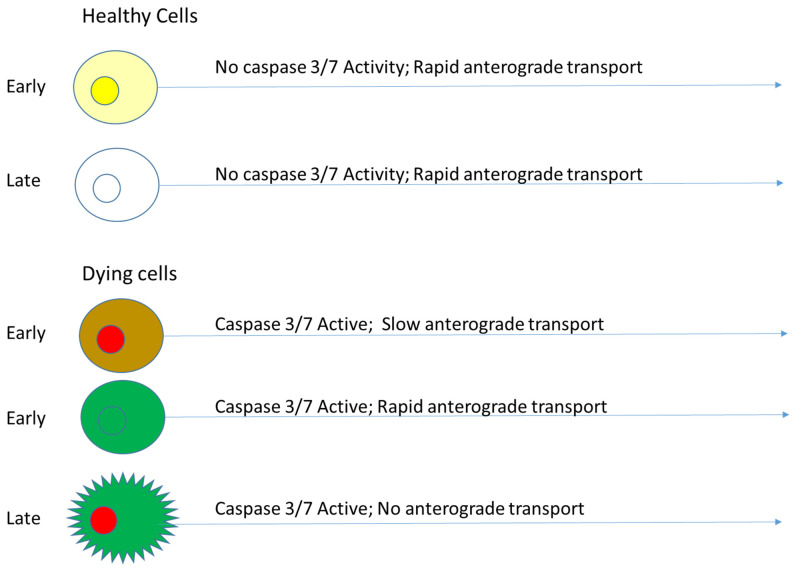
Model of KcapTR488 reporter activation by RGCs in vivo.

In addition to FRET readouts with KcapTR488, the sensor was amenable to quantitative analysis by dual-color localization. By this model, in healthy retinas in vivo, intact KcapTR488, like intact KcapQ488 and TcapQ488 [11,13,18,22,25,26,27], initially enters the nucleus due to the NLS moiety (appearing yellow) but may be transported away from the cell body by anterograde transport down the axon, out of the eye, and out of the field of view using machinery that also recognizes cationic NLS moieties [28]. However, when cells begin to apoptose, activated caspases 3/7 release the NLS-Texas Red and C-terminus AF488 fragments, allowing the cell cytoplasm to become green (Figure 5). Subsequently, as anterograde transport is slowed and eventually halted, the NLS-Texas Red accumulates in the nucleus, yielding red nuclei with green cytoplasm. As the cell membrane and nuclear membranes disintegrate, all species begin to leak out of the cell at various rates.

NMDA-mediated apoptosis was utilized to characterize KcapTR488 in vivo using **ex vivo** fluorescence microscopy. After intravitreal injection, neurons in the GCL showed robust KcapTR488 uptake. The identity of these cells was confirmed by multicolor fluorescence imaging, as the cells were primarily RGCs but also included displaced amacrine cells. Note that while displaced amacrine cells in the GCL are common in rodents, in diurnal Anthropoidea, including humans, these cells are less prominent in the GCL [26]. As anticipated, in healthy/untreated retinas, once the reporter was intracellular, the NLS guided the reporter both towards the nuclei as well as the axonal anterograde transport machinery. When the sensor was intact, both Texas Red and AF488 were enriched in the nuclei. Therefore, the nuclei were qualitatively yellow. When the reporter was cleaved by caspases 3/7 in an apoptotic cell, only the Texas Red fragment was retained in the nucleus or free to be transported down the axon. The AF488 fragment resided within the cytoplasmic pool and was subject to normal slow exchange into the axon and nucleus. Thus, instead of yellow, the nuclei subsequently became red, while the free AF488 fragment localized to the cytosol. A full separation of red and green signals in RGCs took up to 24 h to form in vivo under the model of NMDA-induced RGC apoptosis. At intermediate time points, interesting transition states were observed. In some cases, green cell bodies were observed. This indicated that after the reporter had been cleaved over time, the NLS-Texas Red had been transported out of the nucleus and the cell body, down the axon, and out of the eye. This further suggested that green-labeled cells maintained significant functional axonal transport, even after the effector caspase enzyme had been activated, or that other populations of cells have a predisposition to an early loss of axonal transport during NMDA-mediated cell death. For those interested in identifying and studying these subsets, KcapTR488 could be of significant utility.

While qualitative analyses are important and informative, quantitative measurements are less subject to intra-observer and -operator variation. By design, changes in the subcellular localization of the NLS-Texas Red would be expected to reduce the correlation between the red and green channels. Furthermore, as demonstrated by the in vitro data, the cleavage of the DEVD sequence would be expected to yield changes in the pixelwise red/green ratio through decreased FRET. Indeed, the quantification of the colocalization could be achieved by Manders’s overlap coefficient analysis [17]. The overlap coefficient is much less sensitive to changes in channel brightness than the Pearson correlation coefficient. Both the M1 and M2 values showed significant trends among experimental conditions from healthy untreated eyes to NMDA-treated eyes analyzed 2, 6, or 24 h after the injection of the reporter (Figure 3I,J). Furthermore, there was sufficient precision to detect the difference between NMDA-treated and untreated eyes as early as 2 h after the injection of the reporter (*p* < 0.0001, d = 14). When compared to the FRET ratio, Manders’s overlap coefficient was more robust for detecting early changes in the apoptotic progression in the NMDA model of RGC death. Unlike analysis in cellulo, in which there is a closed homogenous compartment that contains all starting materials and products, a variety of transport and leak processes in vivo appear to render FRET less robust for analysis than changes in colocalization. For example, a loss of an AF488-cysteine product from the field of view or pixel that also retains intact reporter would result in an artificial net increase in the FRET efficiency and would therefore be interpreted as a “loss” of sensor activation and cell death, an incorrect conclusion both quantitatively and directionally. Additionally, the loss of both an AF488 product and an intact peptide with a rapid increase of TR fragment (>10 X) would also yield an artifactual increase in the FRET ratio. Other techniques such as hyperspectral fluorescence unmixing or 4–5 channel fluorescence unmixing might help disambiguate these cases by assigning an intensity and potentially concentration to each fragment. However, the same loss of AF488 fragment, or the loss of AF488 product and intact peptide, would not affect directionality of the overlap coefficient. Indeed, the loss of AF488-cysteine from the field of view would only appropriately enhance the change in Manders’s overlap coefficients, leading to the correct conclusion of apoptotic cell death.

While leveraging the M1 and M2 overlap ratios at the whole-field level was superior to FRET at the whole-cell level, data suggest that this may not be the case if an appropriate FRET and multichannel scanning confocal ophthalmoscope were developed and commercialized for in vivo imaging at the mesoscopic scale. Such an instrument would allow researchers to take advantage of this reporter to exploit at least two different but biochemically related imaging metrics for an analysis arising from a single chemical entity. Due to the underlying nonhomogeneous and fractal nature of biological systems, all spatiotemporal data sets suffer from a special case of Simpson’s paradox [27,29], whereby when different scales and resolutions are tested, new and reversing correlations can be found. Importantly, both are equivalently “true”, and neither are false. Thus, for example, at one scale with one acquisition system, overlap signals may be superior, while at another scale and another resolution, FRET may become superior. While new statistical methods are always under development to mitigate this risk [30,31], these properties are often emergent at each change in scale/support. Thus, sensors that offer multiple readouts at multiple scales, such as the one reported herein, may be more robust as a single chemical entity to changes in scales/instrumentation, potentially facilitating their eventual broad adoption.

As consistently found with four previous generations of molecular beacon reporters, the change in subcellular compartmentalization also corresponded with TUNEL staining in a cross-sectional analysis (Figure 4). An analysis of KcapTR488 cleavage, and therefore caspase activation, might also be studied with multichannel fluorescence correlation spectroscopy (FCS), particularly as studied by the change in the photon-counting histogram (PCH), which is intuitively related to the overlap coefficient herein, except on the single-molecule scale [32,33]. However, such a demonstration was outside of the scope of this project.

KcapTR488 has been extensively characterized in vitro and in vivo. The in vitro results demonstrated resonance energy transfer between AF488 and Texas Red, enabling a conventional ratiometric characterization of caspase cleavage efficiency and quantitative analysis through overdetermined spectral unmixing. The in vivo studies also confirmed the delivery of KcapTR488 to the GCL neurons, while quantitative changes in the colocalization of the fluorophores Texas Red and AF488 readily revealed the expected time-dependent activation of cell death following NMDA treatment. Unexpectedly, a quantitative analysis of changes in colocalization proved superior to a quantitative analysis of changes in the FRET ratio for differentiating NMDA-induced toxicity from healthy eyes as early as 2 h after the injection of the reporter.

## Figures and Tables

**Table 1:** Summary of biochemical characterization of KcapTR488.

Caspase	Class	K_m_ (μM) (SEM)	K_cat_ (1/s) (SEM)	Efficiency (1/(s × M) (SEM)
3	Executioner	2.2 (0.4)	0.59 (0.1)	30 × 10^4^ (9.1 × 10^4^)
7	Executioner	1.5 (0.5)	0.005 (0.001)	0.3 × 10^4^ (0.05 × 10^4^)
8	Initiator	5 (5)	0.03 (0.02)	1.0 × 10^4^ (0.5 × 10^4^)
1	Inflammatory	2.5 (0.09)	0.01 (0.0005)	0.48 × 10^4^ (0.005 × 10^4^)

## Data Availability

All data are contained within this report.

## Supplementary Materials

The following supporting information can be downloaded at: https://www.mdpi.com/article/10.3390/bios12090693/s1, Figure S1: Emission spectra of KcapTR488, Alexa488 and Texas Red; Figure S2: An equation derived to measure the amount of product in the biosensor FRET experiment [34]; Figure S3: Caspase cleavage independently validated by HPLC analysis.
