# Supplementary material for: Apoptosis Detection in Retinal Ganglion Cells Using Quantitative Changes in Multichannel Fluorescence Colocalization
Source: Biosensors (Basel). 2022 Aug 28;12(9):693. doi: 10.3390/bios12090693 (PMC9496076; doi:10.3390/bios12090693)
Supplement: Supplementary file 1 [file biosensors-12-00693-s001.zip › biosensors-1808371-supplementary.pdf]

biosensors

MDPI

Article

Apoptosis Detection in Retinal Ganglion Cells Using Quantitative Changes in Multichannel Fluorescence Colocalization

Xudong Qiu [1,†], Seth T. Gammon [1,†], James R. Johnson [2], Federica Pisaneschi [1], Steven W. Millward [1], Edward M. Barnett [3], and David Piwnica-Worms [1,*]

[1] Department of Cancer Systems Imaging, University of Texas MD Anderson Cancer Center, Houston, TX 77030, USA

[2] Mallincrodt Institute of Radiology, Washington University in St. Louis, St. Louis, MO 63130; Current affiliation: MediBeacon, St. Louis MO 63141, USA

[3] Department of Ophthalmology & Visual Sciences, Medical College of Wisconsin, Milwaukee, WI 53226, USA

* Correspondence: dpiwnica-worms@mdanderson.org.

† These authors contributed equally to this work.

Supplementary Materials: The following supporting information can be downloaded at: www.mdpi.com/xxx/s1, Figure S1: Emission spectra of KcapTR488, Alexa488 and Texas Red; Figure S2: An equation derived to measure the amount of product in the biosensor FRET experiment; Figure S3: Caspase cleavage independently validated by HPLC analysis.

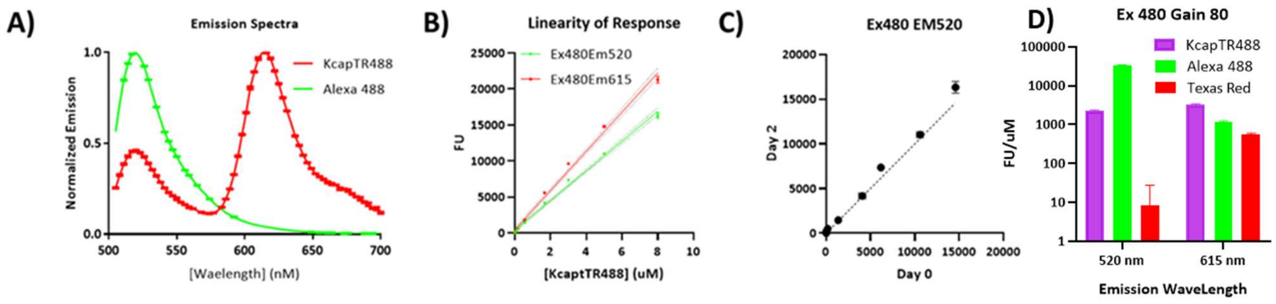

Figure S1. Emission spectra of KcapTR488, Alexa488 and Texas Red. Emission spectra of KcapTR488 and Alexa488 in caspase buffer normalized to max value (error bars, SEM) (A). Dilution series of KcapTR488 is linear at key excitation and emission wavelengths; dotted lines represent 95% CI for regression; error bars represent SEM and may be obscured by the size of the datapoint because they are so small over the range tested; R^2 > 0.99 (B). The fluorescent response is highly reproducible from day to day (dotted line = perfect agreement) over the range of peptide concentrations utilized in the experiment (C). The spectral response for key fluorophores were measured for KcapTR488, Alexa488, and Texas Red (D).

Reaction scheme KcapTR488 + Caspase 3 -> TexasRed Fragment + Alexa488 Fragment

1) [KcapTR488]=½[Alexa488f]+½[TexasRedf]– mass in a closed system is conserved
2) [Alexa488f]=[TexasRedf]=[Product] from chemical reaction above
3) $[KcapTR488]_t=[KcapTR488]_i-[Product]_t$ from reaction above at the concentration of kcaptr448 is the initial concentration minus the concentration of either product formed
4) $EM520 = [KcapTR488]_t B^{520}_{KcapTR488}+[Alexa488f]_t B^{520}_{488}+[TexasRedf]_t B^{520}_{TR}$ The amount of fluorescence is the sum of the concentration of each reagent times its Molar response co-efficient
$EM615 = [KcapTR488]_t B^{615}_{KcapTR488}+[Alexa488f]_t B^{615}_{488}+[TexasRedf]_t B^{615}_{TR}$
5) $EM520 = [KcapTR488]_t B^{520}_{KcapTR488}+[Product]_t B^{520}_{488}+[Product]_t B^{520}_{TR}$ from 2
$EM615 = [KcapTR488]_t B^{615}_{KcapTR488}+[Product]_t B^{615}_{488}+[Product]_t B^{615}_{TR}$
6) $EM520 = ([KcapTR488]_i - [Product]_t)B^{520}_{KcapTR488}+[Product]_t B^{520}_{488}+[Product]_t B^{520}_{TR}$ from 3
$EM615 = ([KcapTR488]_i - [Product]_t)B^{615}_{KcapTR488}+[Product]_t B^{615}_{488}+[Product]_t B^{615}_{TR}$
7) $EM520-[KcapTR488]_i * B^{520}_{KcapTR488} = [Product]_t * (B^{520}_{488}+B^{520}_{TR}-B^{520}_{KcapTR488})$ Collect measured terms
$EM615-[KcapTR488]_i * B^{615}_{KcapTR488} = [Product]_t * (B^{615}_{488}+B^{615}_{TR}-B^{615}_{KcapTR488})$
12) $S^W = EM^W-[KcapTR488]_i * B^W_{KcapTR488}$ define a constant S^w for each concentration and emission wavelength tested
13) $Z^W = B^w_{488}+B^W_{TR}-B^W_{KcapTR488}$ Collect Molar response coefficients into a single term for each emission wavelength
14) $S^{520}= [Product]_t * Z^{520}$ substitute based upon 12 and 13
$S^{615}= [Product]_t * Z^{615}$
16) $\mathbf{S}= \mathbf{Z}.[Product]_t$ convert 14 from multiple equations into a single matrix problem
17) $\mathbf{Z}‡.\mathbf{S}=\mathbf{Z}‡.\ \mathbf{Z}[Product]_t$ Leverage the pseudo inverse to solve the overdetermined matrix problem
18) $\mathbf{Z}‡.\mathbf{S}=\mathbf{I}[Product]_t$

Figure S2. An equation derived to measure the amount of product in the biosensor FRET experiment. Given that the *in vitro* system is closed, mass and concentration are conserved. Furthermore, caspases cleave the peptide into two equimolar fragments, one containing Alexa488 (Alexa488f) and one containing Texas Red (TexasRedf). The coefficients $B^{\text{emission wavelength reporter}}$ represent the spectral response for a given fluorophore at a given wavelength as measured in *Figure S1D*. $EM^{\text{wavelength}}$ describes the emission observed in a well in fluorescence units at a given wavelength. Thus, an over determined set of linear equations is generated. $S^{\text{wavelength}}$ represents a collection of constants and measured terms for a given wavelength. $Z^{\text{wavelength}}$ represents a collection of constants at a given wavelength. The converted matrix equation (indicated by bold) can be solved through the pseudoinverse function (double dagger symbol) and applied to each starting concentration of KcapTR488 and at each time point in the experiment to calculate the amount of product (Mathematica v10, Champaign, IL). Thus, cleavable biosensors in closed compartments represent a special more precise case of the general spectral unmixing algorithm for FRET determination [1].

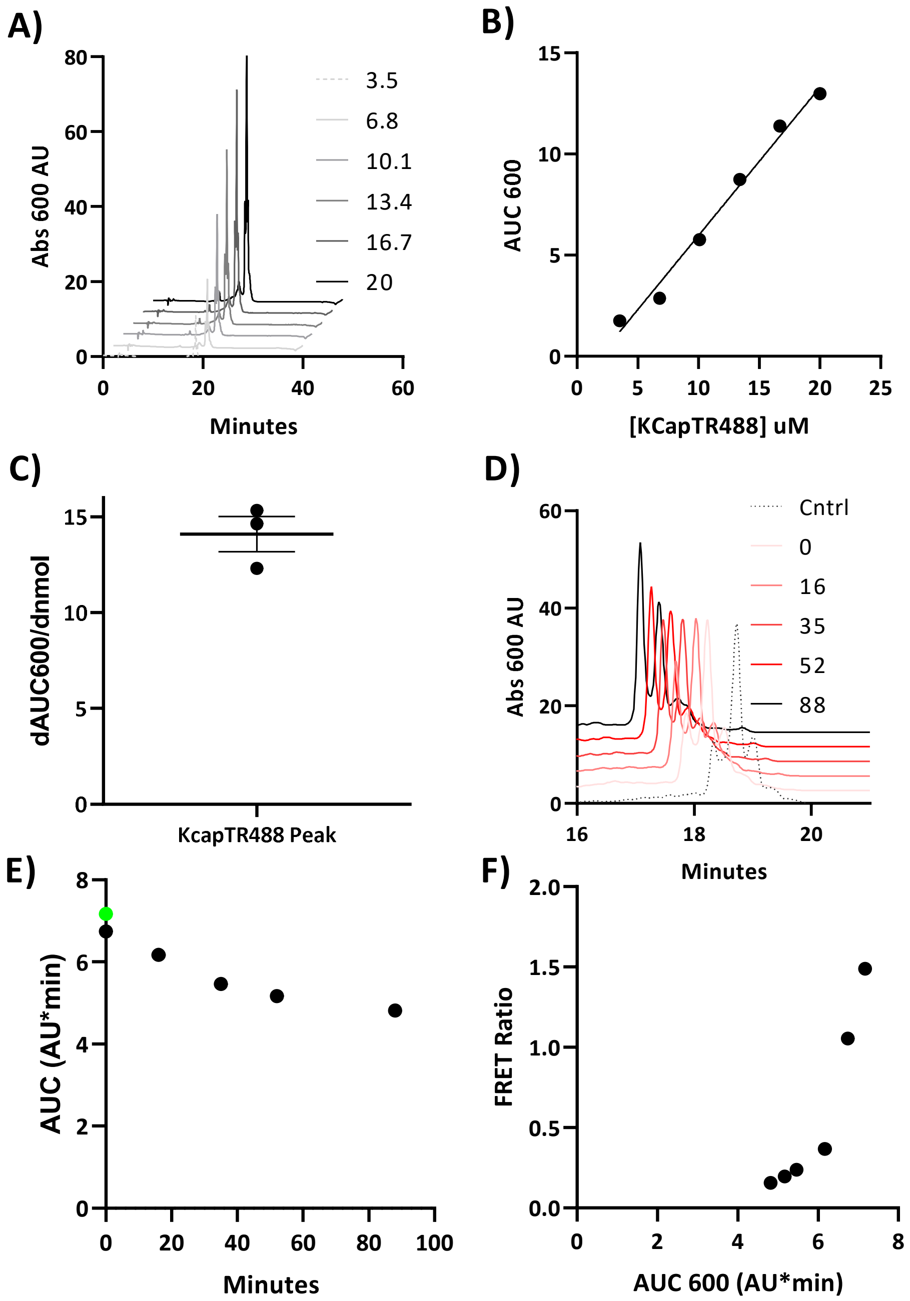

Figure S3. Caspase cleavage independently validated by HPLC analysis. Example of purity and dose response of a linear gradient of KcapTR488 studied via reverse phase HPLC using 600 nm absorbance (A) (HPLC method specifics: Column: Alltech Adsorboshere C18 5u, 250 mm. Method: A- water (0.1% TFA); B – acetonitrile (0.1% TFA); %B 5 → 40 in 15 min, 40 for 15 min, 40 → 95 in 3 min, 95 → 5 in 1 min.) . Both peak height (not shown) and area under the curve of the KcapTR488 primary peak are linear over the range tested $R^2 > 0.98$ (B). Amount injected into HLPC was converted into nanomoles and the slopes were compared across three days, with a COV = 11%, thus suitable for measuring loss of parental peak with cleavage by caspase 3 (C). Caspase 3 (20 nM) was incubated with KcapTR488 (10 μM) for the times (min) indicated. The sample fluorescence was measured, quenched with ethanol and snap frozen. The sample was then thawed, kept on ice and injected for HPLC analysis on the subsequent day. A clear time-dependent loss of the KcapTR488 peak and the emergence of a faster eluting peak consistent with a caspase-shortened peptide occurs (D) (Note: peaks are slightly shifted in the x and y axis for clarity). The area under the curve of the parental peak was quantified as in B&C, and a non-linear decrease in the AUC of the KcapTR488 peak was observed over time. The green point represents a control that had no enzyme incubated for the full 88 min (E). These data confirm that caspase 3 was physically cleaving the peptide into a product, not just inducing a conformational change that changed the FRET measurements. Finally, as expected, the FRET

ratio measured the day prior, positively and non-linearly correlated (r=1, p=0.0028 Spearman r) with the amount of parental product peak present on the HPLC on a per sample basis.

Reference

1. Leavesley, S.J., et al., *Assessing FRET using spectral techniques.* Cytometry A, 2013. **83**(10): p. 898-912.
